# Rapid Antemortem Detection of CWD Prions in Deer Saliva

**DOI:** 10.1371/journal.pone.0074377

**Published:** 2013-09-11

**Authors:** Davin M. Henderson, Matteo Manca, Nicholas J. Haley, Nathaniel D. Denkers, Amy V. Nalls, Candace K. Mathiason, Byron Caughey, Edward A. Hoover

**Affiliations:** 1 Department of Microbiology, Immunology and Pathology, College of Veterinary Medicine and Biomedical Sciences, Colorado State University (CSU), Fort Collins, Colorado, United States of America; 2 Laboratory of Persistent Viral Diseases, Rocky Mountain Laboratories (RML), National Institute of Allergy and Infectious Disease, Hamilton, Montana, United States of America; 3 Department of Biomedical Sciences, University of Cagliari, Monserrato, Italy; University of Maryland School of Medicine, United States of America

## Abstract

Chronic wasting disease (CWD) is an efficiently transmitted prion disease of cervids, now identified in 22 United States, 2 Canadian provinces and Korea. One hallmark of CWD is the shedding of infectious prions in saliva, as demonstrated by bioassay in deer. It is also clear that the concentration of prions in saliva, blood, urine and feces is much lower than in the nervous system or lymphoid tissues. Rapid *in vitro* detection of CWD (and other) prions in body fluids and excreta has been problematic due to the sensitivity limits of direct assays (western blotting, ELISA) and the presence of inhibitors in these complex biological materials that hamper detection. Here we use real-time quaking induced conversion (RT-QuIC) to demonstrate CWD prions in both diluted and prion-enriched saliva samples from asymptomatic and symptomatic white-tailed deer. CWD prions were detected in 14 of 24 (58.3%) diluted saliva samples from CWD-exposed white-tailed deer, including 9 of 14 asymptomatic animals (64.2%). In addition, a phosphotungstic acid enrichment enhanced the RT-QuIC assay sensitivity, enabling detection in 19 of 24 (79.1%) of the above saliva samples. Bioassay in Tg[CerPrP] mice confirmed the presence of infectious prions in 2 of 2 RT-QuIC-positive saliva samples so examined. The modified RT-QuIC analysis described represents a non-invasive, rapid ante-mortem detection of prions in complex biologic fluids, excreta, or environmental samples as well as a tool for exploring prion trafficking, peripheralization, and dissemination.

## Introduction

Chronic Wasting Disease (CWD) is a transmissible spongiform encephalopathy (TSE), or prion disease, that affects free-ranging and captive cervids [1,2]. CWD appears to be the most transmissible of the prion diseases, and is now recognized in twenty-two U.S. states, as well as two Canadian provinces and the Republic of Korea (http://www.nwhc.usgs.gov/). Experimental studies have demonstrated that CWD prions can also infect several outbred non-cervid species [3–9]. Thus the emerging prevalence of CWD poses a challenge to wildlife management agencies, free-ranging and captive cervid populations, the hunting and food producing animal economies, and may pose a zoonotic risk. Ideally, monitoring for CWD prions would be carried out on minimally invasive biologic samples (such as saliva, blood, urine or feces) harvested from live animals in the field. However, rapid, sensitive and specific ante-mortem detection of CWD and other prion diseases remains a challenge due to the low concentrations of prions and the presence of inhibitors in body fluids and excreta [10–13].

The salient feature of prion disease is the conversion of the normal cellular prion protein (PrP^C^) to a misfolded, pathogenic and transmissible form, often designated as PrP^Res^, PrP^Sc^, or PrP^D^ [14–16]. The normal prion protein (PrP^C^), expressed at highest level in the central nervous system [17,18], is composed of ~250 amino acids with a predominantly unfolded N-terminal region and a C-terminal domain that is folded, globular, and contains three α-helices and two short β-sheet stretches [19,20]. The formation of the misfolded pathogenic prion is thought to occur through PrP^Res^-templated conversion of the predominantly α-helical C-terminal region of PrP^C^ to a high β-sheet oligomeric conformer, thereby conferring partial protease resistance [21–23]. The degree of protease-resistance of PrP^Res^ varies markedly from oligomers to large amyloid fibrils, with the smaller particles tending to be more infectious per unit protein [24]. Bioassay studies in deer have demonstrated infectious prions in the saliva, urine, blood and feces of infected deer [25–27]. However, due to the low concentration of PrP^Res^ detectable in biological fluids or excreta and the potential that the infectious prions may be relatively protease-sensitive, the timing, source and biochemical nature of peripheralized/excreted prions remain poorly understood. Thus, detection of the low levels of prion protein in body fluids will likely require *in vitro* amplification such as that provided by serial protein misfolding cyclic amplification (PMCA) [28–31]. PMCA has been very successfully applied to tissue samples, however, detection of prions in some body fluids has been hampered by the presence of inhibitors in biological fluids and excreta. Nevertheless, the detection of scrapie prions by multiple rounds of PMCA performed on oral swab eluates from scrapie-infected sheep [32] and blood of hamsters [29] has demonstrated the potential for detection of very low levels of prions in body fluids through in vitro amplification methods.

Real-time quaking conversion (RT-QuIC) [33,34] relies on the seeded conversion of recombinant PrP^C^ to a thioflavin T (ThT)-binding amyloid-like PrP form and offers the potential for sensitive ante-mortem detection of prions in a single round assay [35,36]. Here we report adaptations of RT-QuIC to detect CWD prions in the saliva of CWD-exposed pre-symptomatic and symptomatic deer. These data support the promise of RT-QuIC methodology both for sensitive prion detection in live animals and as a means to help elucidate the mechanisms of prion conversion, peripheralization and transmission.

## Materials and Methods

### Expression and purification of rPrP

RT-QuIC assays were performed with recombinant Syrian hamster PrP (SHrPrP) encoding residues 90-231 in Pet 41 and expressed and purified as previously described [34]. In brief, 1 liter cultures of LB containing Auto Induction™ supplements (EMD Biosciences) were inoculated with SHrPrP expressing Rosetta strain *E. coli*, grown overnight, and harvested when OD (600 nm) of ~3 was reached. Cells were lysed with Bug Buster™ reagent with supplemented Lysonase™ (EMD Biosciences) and inclusion bodies (IB) were harvested by centrifugation of the lysate at 15,000 x g. IB pellets were washed twice and stored at -80 °C until purification (typically 24 hours or less). IB pellets were solubilized in 8 M guanidine hydrochloride (GuHCl) in 100 mM NaPO_4_ and 10 mM Tris pH 8.0, clarified by centrifugation at 15,000 x g for 15 min and added to Super Flow Ni-NTA resin (Qiagen) pre-equilibrated with denature buffer (6.0 M GuHCl, 100 mM NaPO_4_, 10 mM Tris pH 8.0). Denatured SHrPrP and Ni-NTA resin was incubated by rotating at room temperature for 45 min and then added to an XK FPLC column (GE Healthcare). Refolding was achieved on column using a linear refolding gradient of denature buffer to refold buffer (100 mM NaPO_4_, 10 mM Tris pH 8.0) over 340 ml at 0.75 ml/min. SHrPrP was eluted with a linear gradient of refold buffer to elution buffer (100 mM NaPO4, 10 mM Tris pH 8.0 500 mM imidazole pH 5.5) over 100 ml at 2.0 ml/min. Fractions were pooled and dialyzed against two changes of 4.0 l dialysis buffer [20 mM NaPO_4_ pH 5.5 (CSU); 10 mM NaPO_4_ pH 5.8 (RML)]. SHrPrP was adjusted to ~0.5 mg/ml, aliquoted, snap frozen in liquid nitrogen, and then stored at -80 °C.

### Animals and samples

All animals were handled in strict accordance with guidelines for animal care and use provided by the United States Department of Agriculture (USDA), National Institutes of Health (NIH) and the Association for Assessment and Accreditation of Laboratory Animal Care International (AAALAC), and all animal work was approved by Colorado State University Institutional Animal Care and Use Committee (IACUC approval numbers 02-151A, 08-175A, 09-1463A, 10-1766A, 10-2189A, 11-2622A and 12-3773A).

The CWD-naïve, hand-raised, human- and indoor-adapted white-tailed deer fawns were sourced from the Warnell School of Forestry, University of Georgia as part of longstanding collaborations with David Osborn, Sallie Dahmes, Carl Miller and Robert Warren. The deer were housed in indoor barrier-maintained pens at the Colorado State University CWD Research Facility at the Foothills Campus. As part of CWD transmission, pathogenesis, and vaccination studies, they were exposed to CWD prions contained in various inoculation materials delivered by various routes (Table 1). Saliva samples were collected via syringe aspiration or direct tube collection predominantly from anesthetized deer and stored at -80 °C until thawing and dilution for the assays described. The majority of samples forming the basis of this report were analyzed independently using the same QuIC protocols in the CSU and NIAID/Rocky Mountain laboratories by the first two authors of this report (Table S1). Deer were classified clinically (Table 1) as: (a) pre-clinical—either no symptoms of CWD or behavioral changes sufficiently subtle as to be detectable only by observers experienced with each animal and its demeanor; (b) clinical—varying levels of clinical CWD signs were present, including: weight loss of >10% peak weight, behavioral changes (e.g. postural changes, fixed staring, repetitive motions, aggression or withdrawal), increased frequency of food or water consumption, incoordination, salivation.

**Table 1:** Summary of white-tailed deer sample status and RT-QuIC results.

Animal	Diluted	PTA	Animal	Route	PrP^Res^	Disease State
Number	Saliva	Saliva	Inoculum		IHC^#^	
108	0/8	0/8	CWD(+) Blood	IP/IV	+	Terminal
112	1/8	8/8	CWD(+) Brain	PO	+	Clinical
121	2/8	8/8	CWD(+) Brain	IC	+	Terminal
132	0/8	8/8	CWD(+)Saliva	PO	+	Terminal
133	5/8	8/8	CWD(+) Blood	IV	+	Clinical
136	0/8	6/8	CWD(+) Brain	PO	+	Terminal
137	0/8	4/8	CWD(+) Blood	IV	+	Clinical
138	3/8	10/12	CWD(+) Brain	PO	+	Clinical
143	0/8	1/8	CWD(+) Brain	PO	+	Terminal
144	3/8	8/8	CWD(+)Saliva	PO	+	Terminal
773	0/8	3/8	CWD(+) Brain	PO	+	Pre-Clinical
775	1/8	0/8	CWD(+) Brain	PO	+	Pre-Clinical
776	0/8	8/8	CWD(+) Brain	PO	+	Pre-Clinical
777	1/8	2/12	CWD(+) Brain	PO	+	Pre-Clinical
778	1/8	12/12	CWD(+) Brain	PO	+	Pre-Clinical
780	0/8	1/8	Ovine Scrapie (+) Brain	IV	+	Pre-Clinical
781	0/8	0/8	CWD(+) Brain	PO	+	Pre-Clinical
785	1/8	12/12	CWD(+) Brain	PO	+	Pre-Clinical
812	1/8	0/8	CWD(+) Brain	Aerosol	+	Pre-Clinical
813	0/8	16/16	CWD(+) Brain	Aerosol	+	Pre-Clinical
815	1/8	8/8	CWD(+) Brain	Aerosol	+	Pre-Clinical
816	3/8	0/8	CWD(+) Brain	Aerosol	+	Pre-Clinical
817	1/8	6/8	CWD(+) Brain	Aerosol	+	Pre-Clinical
818	1/8	2/8	CWD(+) Brain	Aerosol	+	Pre-Clinical
810	0/8	0/8	(-) Brain	Aerosol	-	N.A.
814	0/8	0/8	(-) Brain	Aerosol	-	N.A.
819	0/8	0/16	(-) Brain	Aerosol	-	N.A.
103	0/8	0/8	(-) Multiple*	Var. Routes	-	N.A.
123	0/8	0/8	(-) Multiple*	Var. Routes	-	N.A.
502	0/8	0/12	(-) Urine & Feces	PO	-	N.A.
504	1/8	1/8	(-) Urine & Feces	PO	-	N.A.

Summary of animals, inocula and route of infection, sample collection relative to clinical status, and RT-QuIC results. The number of replicates positive from up to 3 experiments is reported for both 1:10 diluted saliva and PTA treated saliva. Definitions: Pre-clinical—either no symptoms of CWD or behavioral changes sufficiently subtle as to be detectable only by observers experienced with each animal and its demeanor; Clinical—varying levels of clinical CWD signs were present, including: weight loss of >10% peak weight, behavioral changes (e.g. postural changes, fixed staring, repetitive motions, aggression or withdrawal), increased frequency of food or water consumption, incoordination, salivation. Inocula abbreviations: intraperitoneal (IP), orally (PO), intravenously (IV), Intracranially (IC). (*) Animals received multiple (-) inocula. Each received CWD (-) blood IP, CWD (-) urine, feces and saliva PO and CWD (-) white-tailed deer brain IC.

### Diluted saliva RT-QuIC assay

The substrate for seeded RT-QuIC reactions was prepared by adding SHrPrP to RT-QuIC reaction buffer composed of 50 mM NaPO_4_, 320 mM NaCl, 1.0 mM ethylenediaminetetraacetic acid tetrasodium salt (EDTA), 10 µM Thioflavin T (ThT, Sigma) to a final concentration of 0.1 mg/ml. The substrate was added to optical bottom black 96-well plates (Nunc) with either 98 µl for brain homogenate samples or 95 µl for 1:10 dilution (Dilution buffer: 0.1% SDS in PBS pH 7.4) saliva samples. 2 µl of CWD positive or negative control brain samples diluted from 10^-4^ to 10^-8^ were added to substrate on each plate. Five µl of 1:10 diluted saliva was added in quadruplicate to substrate wells for each assayed saliva sample. Each saliva sample was assayed in quadruplicate wells at least twice using independently set up substrate reaction mixes. Prepared plates were placed in a BMG Labtech Polarstar™ fluorometer and subjected to 700 rpm double-orbital shaking for one minute every other minute for 15 minutes. After each shaking cycle ThT fluorescence was read at an excitation of 450 nm and emission of 480 nm. Gain was set at 1700 for 1:10 dilution saliva samples and read using orbital averaging with 20 flashes per well with the 4 mm setting. The substrate was replaced after 24 hours by removing 100 µl from each well. A residual amount of liquid remained in each well and a high percentage of the ThT fluorescence. Fluorescent readings were recorded for all sample reactions for a total time of 48 hours at a temperature of 42 °C.

### PTA saliva RT-QuIC assay

In order to concentrate CWD prions and avoid the assay inhibition factors in saliva, 100 µl of each sample was shaken for 1 hour at 1500 rpm and 37 °C (Eppendorf Thermomixer R) in the presence of 0.28% (w/v) phosphotungstic acid (PTA, Sigma) at pH 7.4. After a 30-min spin at 16100 x g at 23 °C, the pellet was resuspended in 10 µl of 0.1% (w/v) SDS/PBS and used as seed for the RT-QuIC reaction.

RT-QuIC reaction substrate composition was as follows: 10 mM phosphate buffer (pH 7.4), 300 mM NaCl, 0.1 mg/ml SHrPrP, 10 µM ThT, and 1 mM EDTA. Ninety-eight µl of substrate was loaded into wells of a black 96-well plate with a clear bottom (Nunc) and seeded with a 2 µl seed sample for a final reaction volume of 100 µl. All reactions contained equivalent final concentrations of SDS (0.002%). The plates were sealed with a plate sealer film (Nalgene Nunc International), and then incubated in BMG Labtech FLUOstar™ plate reader at 46 °C for 65 hours with cycles of 1 min shaking (700 rpm double orbital) and 1 min rest throughout the incubation. ThT fluorescence measurements (450 +/-10 nm excitation and 480 +/-10 nm emission; bottom read, 20 flashes per well, manual gain of 2300, and 20 ms integration time) were taken every 45 min.

### Mouse inoculation studies

Four milliliters of saliva was collected from two experimentally exposed white-tailed deer (deer #133, 12mos post-inoculation, and 144, 27mos post-inoculation) and one sham exposed white-tailed deer (123). Saliva samples were lyophilized using a LabConco lyophilizer, resuspended in 0.4 ml of PBS and dialyzed against PBS to return the samples to isotonicity. Four milliliters of saliva from a negative control deer (deer 123) was prepared concurrently in a similar fashion. Three groups of Tg[CerPrP] 5037 mice [37] (n=9 mice/group) were sedated using an intraperitoneal (IP) injection of 500 µl of ketamine (10 mg/ml) and xylazine (1 mg/ml), and inoculated intracranially (IC) with 30 µl of the concentrated saliva preparation. Each group was inoculated with concentrated saliva from a single source animal (#133, 144, or 123). Following inoculation, mice were monitored daily over the course of disease for evidence of prion infection (incoordination, weight loss, etc.) and humanely euthanized when necessary. Following euthanasia, the left hemisphere of the brain was removed and analyzed for the presence of PrP^Res^ by western blotting.

### Western blotting

The left hemisphere of each cervid brain was divided into 8 rostral to caudal sections for analysis by western blotting. The most rostral region of all 8 slices was prepared as a 10% homogenate suitable for western blotting. Each sample was processed with and without proteinase K (PK; 30 µg/ml) addition for 30 min at 37 °C shaking at 850 rpm. After PK treatment samples were electrophoresed in 12% SDS-PAGE gel (Biorad), transferred to PVDF with the Trans-Blot Turbo semi-dry transfer apparatus (Biorad). Membranes were processed with the Snap-ID™ western blot instrument (Millipore), blocked with Snap-ID blocking reagent (Millipore), and probed with BAR-224-HRP conjugated antibody at a concentration of 1:5000. HRP blots were developed with Pierce ECL-plus™ reagent (Thermo Scientific) and imaged on an Image-Quant LAS-4000™ imager (GE Scientific).

### End-point dilution analysis

End-point dilution analysis was performed as follows. Two microliters of 10^-7^, 10^-8^ and 10^-9^ CWD-positive and 10^-7^ normal deer brain homogenate in dilution buffer were loaded into the RT-QuIC reaction. For each dilution, 12 replicates wells were seeded. Three aliquots (100 µl) of each CWD negative and positive brain homogenate dilutions were PTA precipitated and PTA pellets resuspended in 10 µl of 0.1% (wt/vol) SDS/PBS; 4 wells were seeded with 2 µl of each PTA pellet. RT-QuIC and PTA precipitation were performed as described in PTA saliva RT-QuIC paragraph, Materials and Methods section.

## Results

### RT-QuIC can detect CWD prion seeds in a 1:10 dilution of saliva

**Figure 1:**
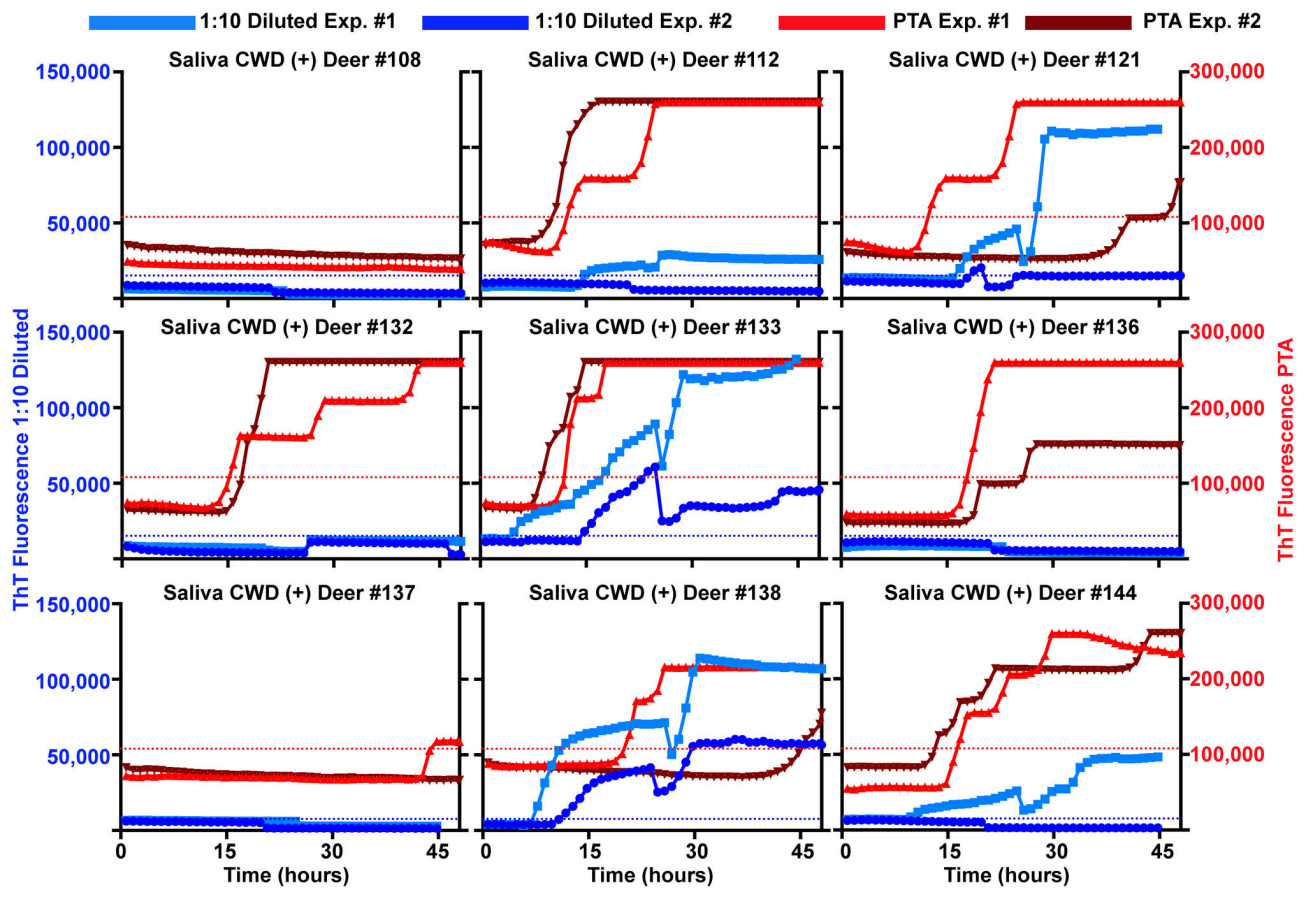
RT-QuIC analysis of CWD-positive clinically ill white-tailed deer. Both 1:10 diluted and PTA precipitated saliva show positive results by RT-QuIC. Each line represents an individual experiment with the average of four replicates. Blue and light blue lines represent 1:10 diluted saliva experiments and red and dark red lines represent PTA precipitated counterpart of the same saliva samples. The left axis (blue) denotes ThT fluorescence for 1:10 diluted saliva samples and the right axis (red) denotes ThT fluorescence for PTA saliva samples. Dashed lines represent threshold for a positive sample. Blue for 1:10 diluted saliva experiments and red for PTA saliva experiments. Positive RT-QuIC results were observed for saliva from clinically ill white-tailed deer in 5 of 9 1:10 diluted saliva samples and 8 of 9 PTA precipitated saliva samples. Each saliva sample was from an individual deer.

**Figure 2:**
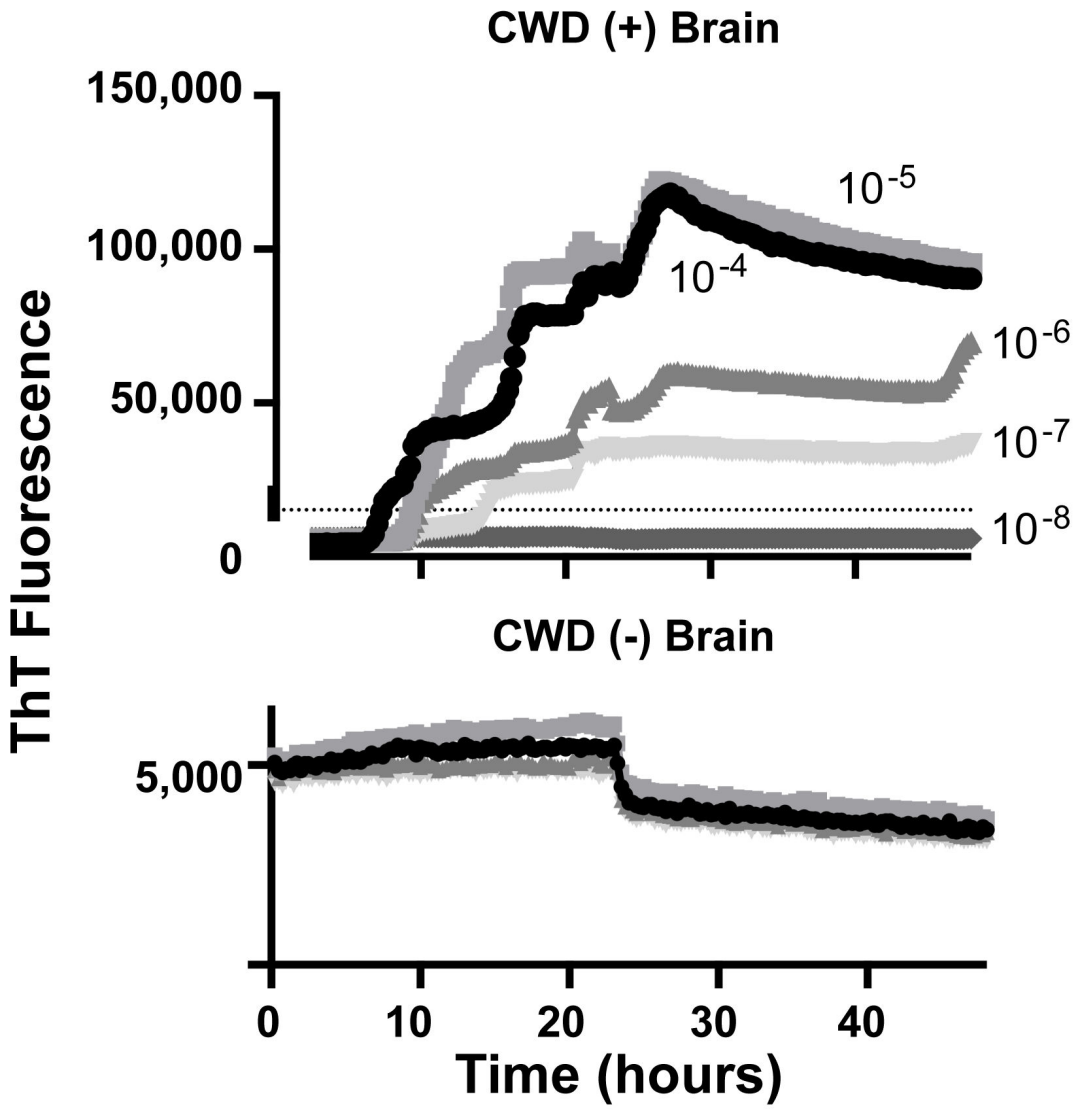
SHrPrP substrate can detect CWD-positive brain over a range of dilutions from 10^-4^ to 10^-7^. CWD positive and negative brain was serially diluted and analyzed by RT-QuIC assay. Each line for positive CWD samples represents the average of 4 individual wells from 4 independent experiments.

Our first goal was to employ the RT-QuIC method for detection of CWD prions in diluted saliva from CWD-inoculated deer. We initially assayed saliva samples diluted from 10^-1^ to 10^-3^. No sample diluted past 10^-1^ yielded a positive result in the RT-QuIC assay, inferring, consistent with previous bioassay studies, a relatively low concentration of prions relative to tissue sources. Nevertheless, we identified a number of saliva samples which at 1:10 dilution in dilution buffer were routinely positive with RT-QuIC. In each of two experiments multiple replicates of these saliva samples were positive with magnitudes and kinetics equivalent to 10^-6^ to 10^-8^ dilutions of CWD positive brain homogenate (Figures 1 and 2). Saliva samples were deemed positive if the average end-point ThT fluorescence of four replicate wells was 3 standard deviations greater than the average fluorescence of the negative control saliva samples. The threshold for positivity with 1:10 diluted saliva, and for PTA-precipitated saliva (see below), was calculated based on the negative controls performed for each method. However, diluted saliva samples from 5 of 10 animals that were known to have clinical CWD symptoms and tonsil or rectal lymphoid tissues positive for CWD PrP^Res^ by immunohistochemistry or western blot were not positive by RT-QuIC (Figure 1 and Table 1). While this could represent the heterogeneity inherent in single time point saliva collections, we nevertheless sought to enhance the salivary RT-QuIC sensitivity without introducing complex concentration methods.

### PTA/RT-QuIC enhances detection of CWD prion seeds in saliva

**Figure 3:**
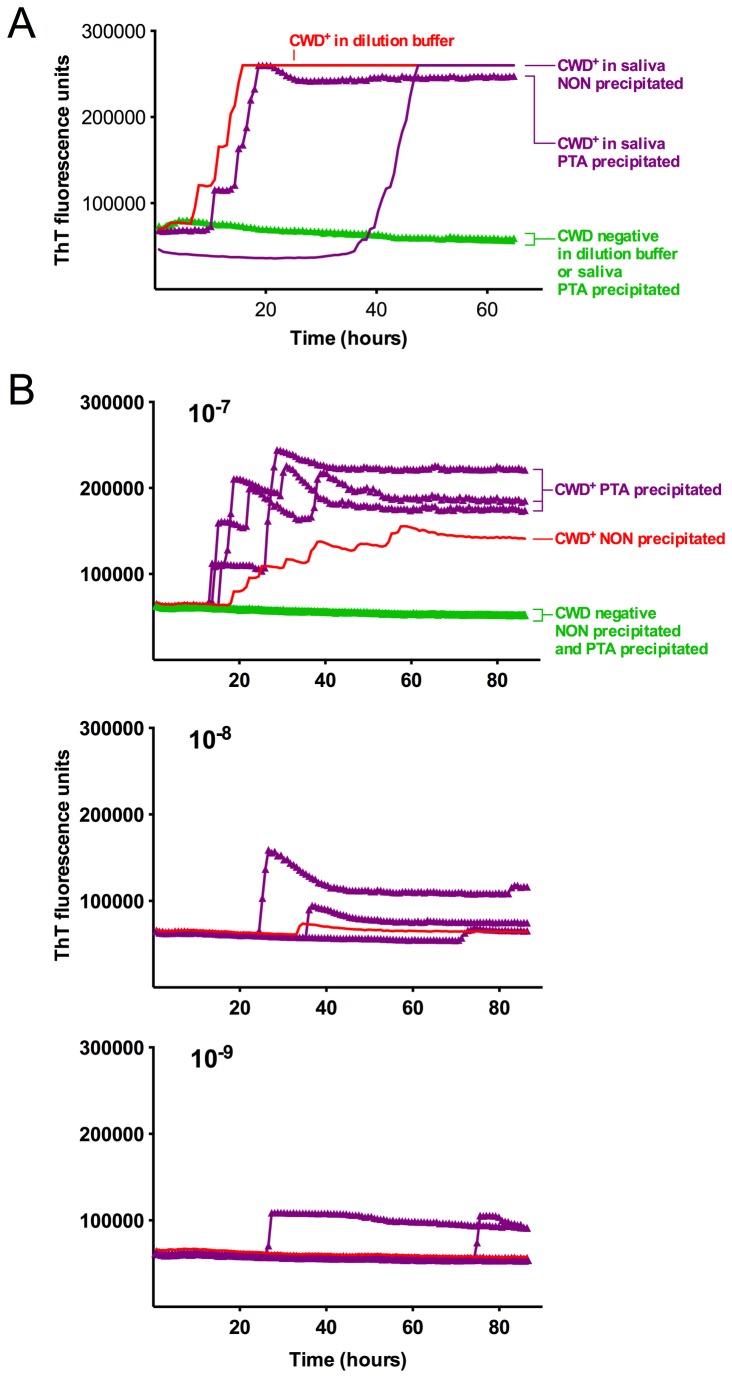
PTA precipitation increases sensitivity of the RT-QuIC assay. A. Each data point shows the ThT fluorescence average of 4 replicate wells loaded with either 2 µl of brain homogenate dilution or PTA pellets from the same brain homogenate equivalents. Lines with triangles represent PTA precipitated samples while smooth lines are non-precipitated counterparts or controls (in the latter case, invisible under the green triangles). The data for spiked PTA precipitated deer saliva (purple triangles) comes from a separate experiment performed under the same experimental conditions. B. End-point dilution analysis of a CWD (+) brain homogenate including 10^-7^ to 10^-9^ dilutions with or without PTA precipitation. PTA precipitated samples were done in triplicate (purple) and represent the ThT fluorescence average of 4 replicate wells each. Non-precipitated samples (red) represent the ThT fluorescence average of all 12 replicate wells. Normal deer brain homogenates (green) are shown for PTA precipitated (3 overlapping curves of data points representing the average of 4 replicate wells) and non-precipitated (average of 12 replicate wells) samples.

With the goal of enhancing the sensitivity of CWD prion detection in saliva (~50% in known infected deer, Table 1) without compromising specificity or introducing cumbersome methodologies, we added pre-assay phosphotungstic acid (PTA) precipitation of saliva samples to the RT-QuIC protocol. PTA precipitation of CWD positive brain spiked into deer saliva samples decreased the lag phase of RT-QuIC reactions (Figure 3A) and increased sensitivity by approximately one order of magnitude (Figure 3B). None of the wells seeded with normal deer brain homogenate or their 3 independently PTA precipitated counterparts gave ThT positivity, showing that the unseeded SHrPrP was stably non-amyloidogenic within the RT-QuIC conditions we used (NaCl 300-320 mM, 42-46 °C, 65 hrs) and that the PTA precipitation protocol per se did not lead to false positive results (Figure 3 A and B). PTA precipitation also reduced spontaneous conversion seen with some undiluted negative control saliva samples, potentially by eliminating or reducing reaction competitors or inhibitors found in saliva, blood and urine. With the addition of PTA precipitation, the same saliva samples from the above group of clinically ill animals retained the same specificity and increased sensitivity from 50 to 90% (5/10 to 9/10) (Figure 1 and Table 1). Both RT-QuIC assay methods showed only a single apparent false positive replicate out of 56 for 1:10 diluted samples and 1 of 68 for PTA precipitation. Each method detected the false positive in the same saliva sample, which likely reflects a predisposition of that sample to cause spontaneous conversion due to contaminants such as rumen or fomites.

### RT-QuIC-positive saliva samples contain infectious prions

**Figure 4:**
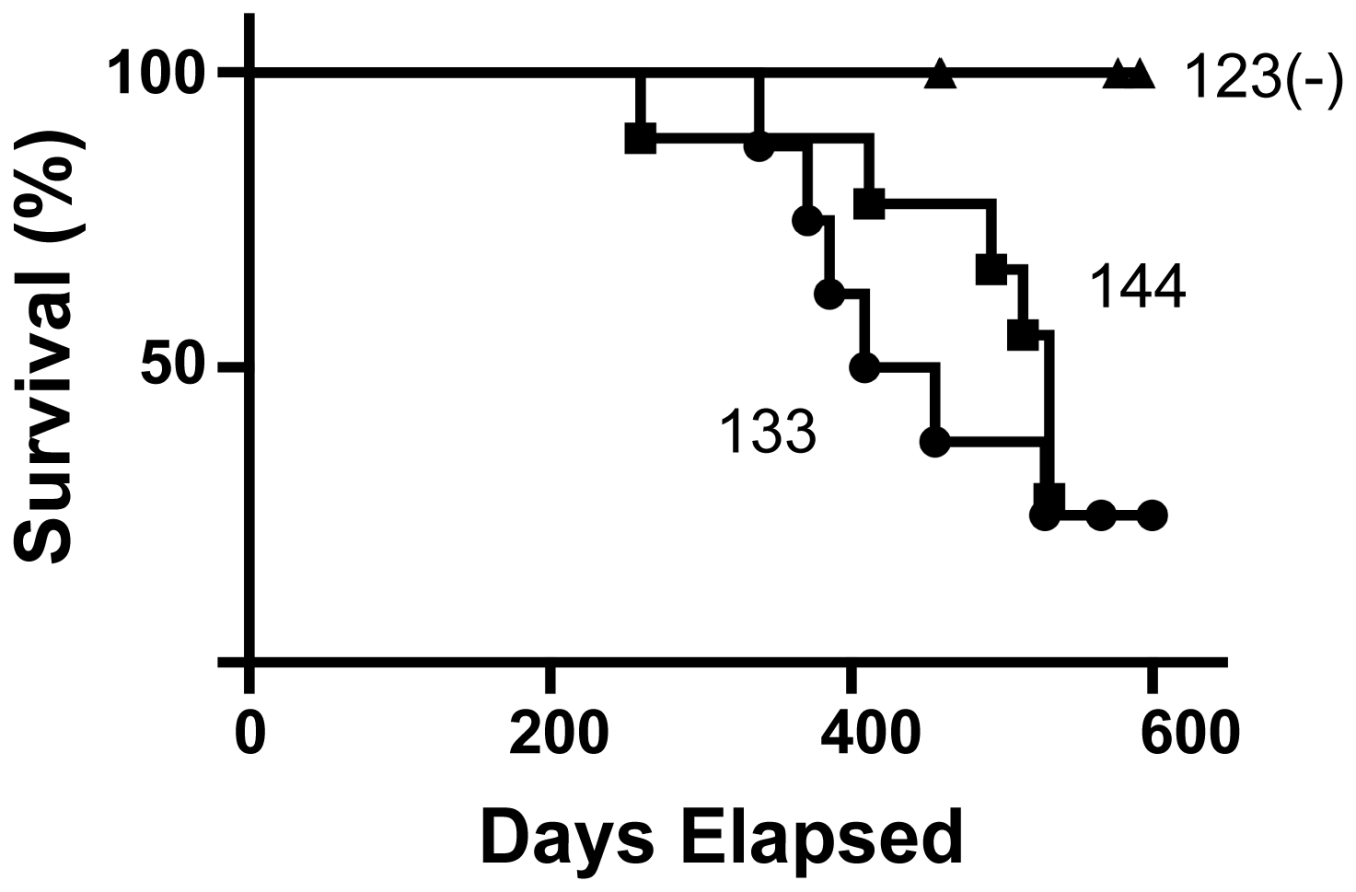
Kaplan-Meier survival curve for mice inoculated with CWD+ saliva. The same saliva samples from deer assayed in Fig. 1 (#133 and 144) were inoculated into cervidized mice (Tg[CerPrP] 5037) as well as saliva from a sham inoculated deer. 50% of mice were euthanized due to clinical disease after ~500 days.

To establish that saliva samples positive for CWD prion seeds by RT-QuIC assay contained authentic infectious CWD prions, transgenic mice expressing normal cervid PrP^C^ (Tg[CerPrP] 5037 mice) [37] were inoculated intracranially with the same saliva samples tested by RT-QuIC from two of the above deer (#133 and 144 from Figure 1). Mice inoculated with each positive saliva sample developed clinical symptoms of TSE as early as ~300 days post-inoculation (dpi) and 50% of the mice were euthanized due to signs attributed to prion infection by 500 dpi, as confirmed by western blotting for PrP^Res^ (Figures 4 and S1). Mice inoculated in the same manner with saliva from an uninfected animal did not develop clinical TSE symptoms nor was PrP^Res^ detectable by western blot (Figures 4 and S1).

### RT-QuIC-based detection of prion shedding in pre-clinical deer

**Figure 5:**
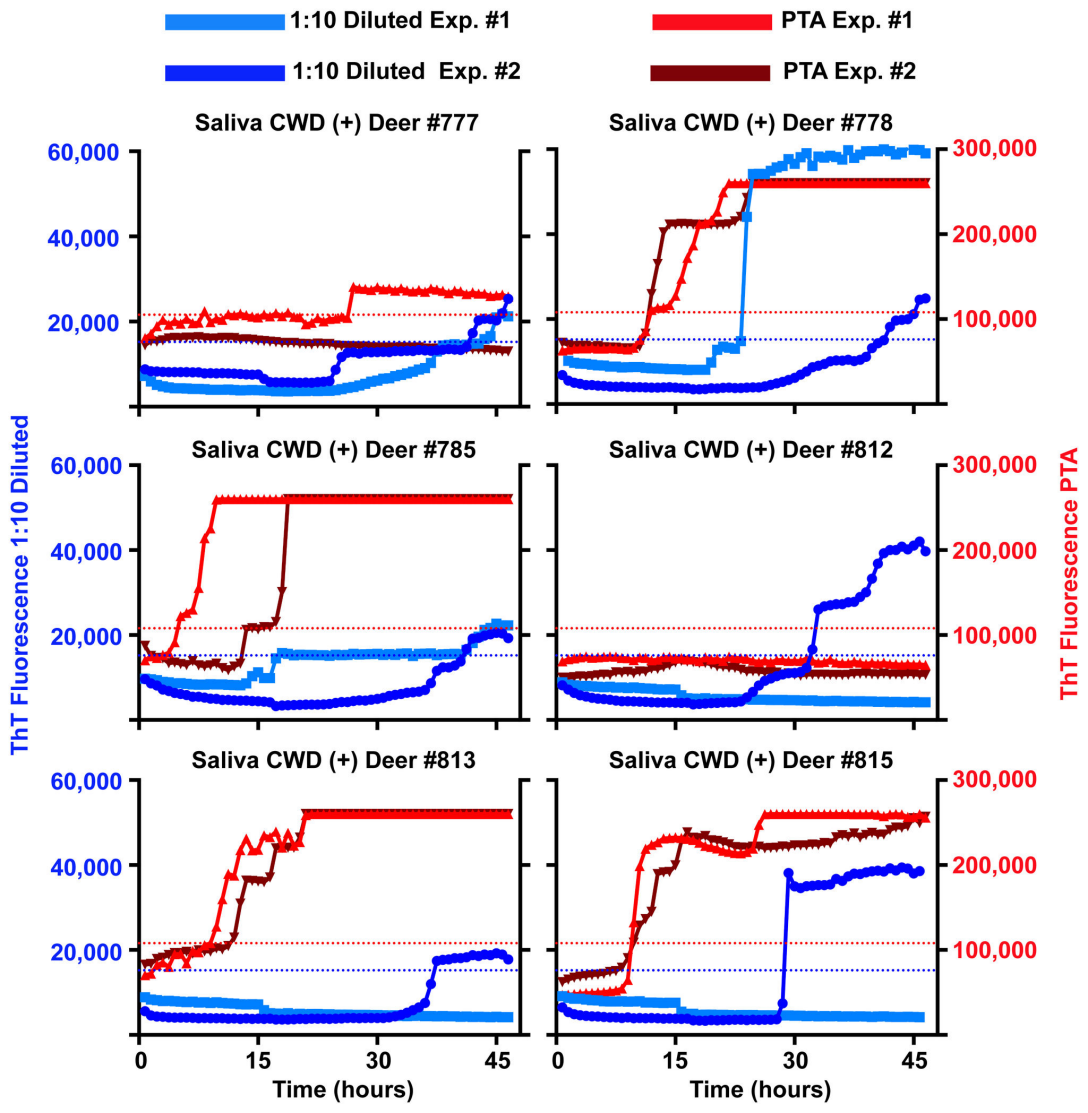
RT-QuIC comparison of diluted saliva vs. PTA precipitated saliva harvested from pre-clinical* white-tailed deer. PTA precipitation of saliva samples increased sensitivity and consistency of RT-QuIC prion detection of pre-clinical white-tailed deer. All colors and axis are the same as in Figure 1. (*) refers to either no signs of CWD or subtle behavioral symptoms detectable only by observers very familiar with individual deer and the very early signs of CWD.

One of our research goals was to establish/develop a non-invasive means to monitor the peripheralization and excretion of prions throughout the CWD disease course. Using parallel analysis of the same saliva samples tested by both 1:10 dilution and PTA-precipitation from pre-clinical CWD-inoculated white-tailed deer in two laboratories, we detected CWD prion shedding in 64.2% of 1:10 diluted samples and 71.4% of PTA precipitated samples (Figure 5 and Table 1). PTA increased the number of positive well replicates from 9.8% with 1:10 dilution to 53.0% and decreased the time for ThT fluorescence to cross the threshold. However, three samples (deer# 775, 812 and 816) that were not positive by PTA RT-QuIC showed a positive signal in the dilution tested samples (Figure 5 and Table 1). These differences may reflect degradation of protease sensitive material during the PTA protocol since no protease inhibitors are used but more work will be necessary to understand the mechanism.

### Correlation of salivary shedding with magnitude of prion dissemination in brain

**Figure 6:**
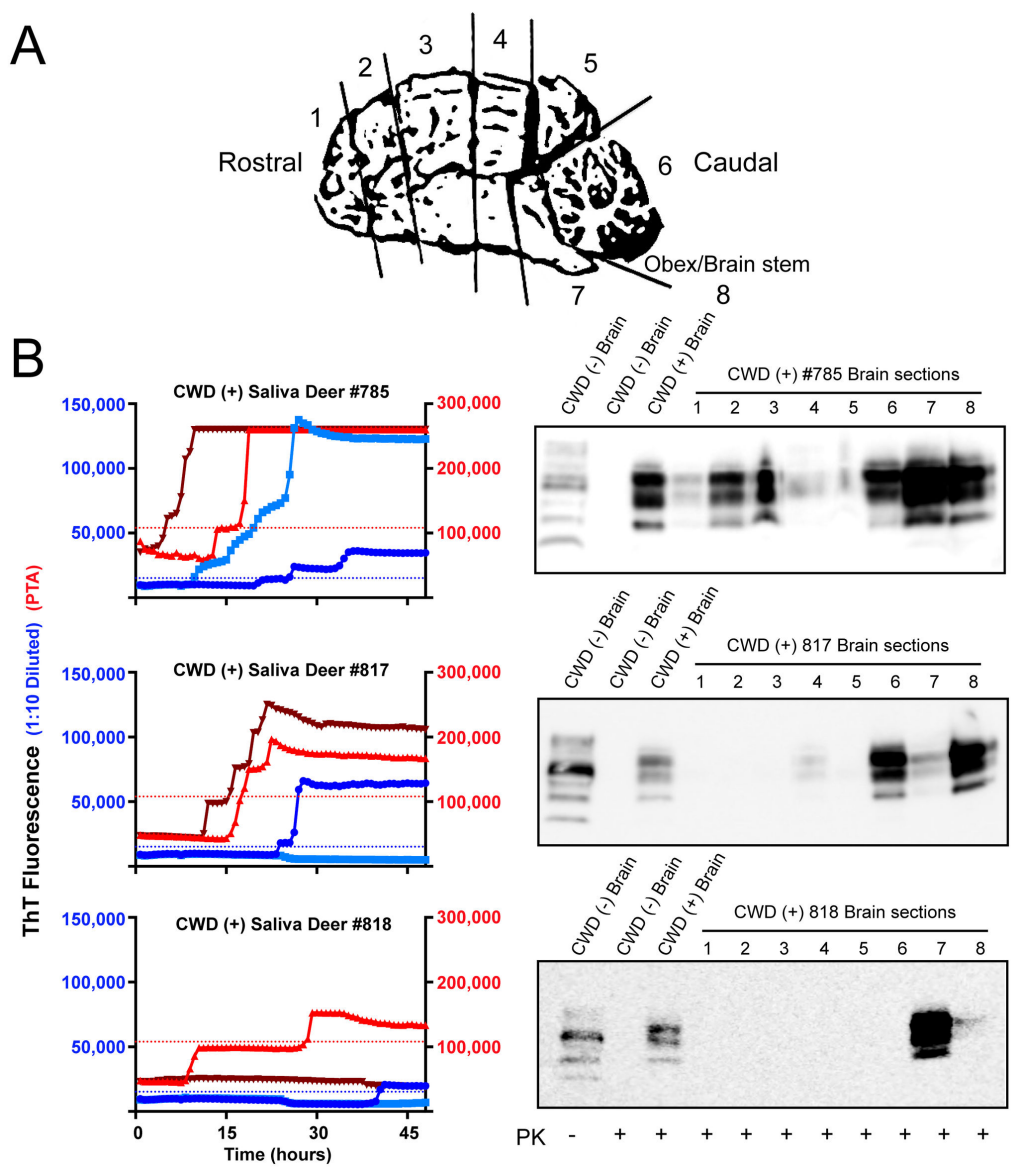
Correlation of RT-QuIC analysis of terminal saliva samples with western blot PrP^Res^ signal in brains. A. Cartoon representation of sections analyzed for PrP^Res^ by western blotting. Seven rostral to caudal sections were analyzed plus the obex/brainstem region. B. (left) RT-QuIC analysis of saliva samples from white-tailed deer. Colors and axis are the same as in Figure 1 (right). Western blot analysis of 8 brain sections including CWD (+) and (-) control samples. PK was added to all samples except one CWD (-) brain sample. Antibody BAR-224-HRP used for detection.

In the course of analyzing both pre-clinical and terminal saliva samples from white-tailed deer, we observed that saliva from some animals produced more robust and consistent positive results by both the PTA and 1:10 diluted RT-QuIC protocols. Because terminal brain was available from most animals, we compared prion dissemination and burden, estimated by western blotting of 8 rostral to caudal brain slices from deer after they had developed terminal disease to RT-QuIC results from the preclinical stage of disease (Figure 6A, B and S2). Widespread distribution and increased magnitude of PrP^Res^ at terminal disease state was often observed for animals with the most RT-QuIC positive saliva replicates during the preclinical stage of disease suggesting a potential correlation between shedding and central nervous system prion load (Figure 6B and S2). Since the western blot data are not quantitative, a strong conclusion regarding direct correlation is not possible, although it would not be surprising that a higher prion load in the brain would lead to higher prion levels shed in saliva.

## Discussion

We demonstrate that RT-QuIC can reproducibly detect prions in saliva, a complex biological fluid containing a wide range of complex glycoproteins including proteases [38–40]. Two of two RT-QuIC-positive saliva samples contained infectious prions by Tg[CerPrP] mouse bioassay and PrP^Res^ distribution in the brain seemed to correlate with RT-QuIC positivity. PTA enrichment of CWD prions in saliva increased the percentage of positive replicates from 17.5% in clinical animals tested by dilution of untreated saliva to 72.6% and from 9.8% in diluted saliva from pre-clinical animals to 53.0%. Previous studies by Colby et al. [41] demonstrated that PTA inhibited amyloid amplification in the amyloid seeding assay. Our results suggest that under the present assay conditions PTA precipitation is not inhibitory but rather increases the sensitivity (Figure 3A and B), perhaps by both concentrating the prions from more saliva equivalents and decreasing saliva-associated inhibitory activities (Figure 3A).

Horizontal transmission of CWD is exceptionally efficient, which may be why it is the only known prion disease found in a free-ranging animal species [42,43]. Enhanced horizontal transmission may reflect abundant excretion and/or unique yet elusive biochemical features of prions shed by CWD infected deer. Multiple studies have demonstrated that relatively protease sensitive and/or oligomeric conformers of PrP^C^ are both infectious and perhaps the more pathogenic species in prion disease [44–48]. Elucidating biochemical differences of prions from saliva or excretory products (e.g. nasal secretions) with the help of the RT-QuIC may reveal possible structural or stability differences between prions in the central nervous system and those shed into the environment.

Several studies indicate that the concentration of prions in excreta is very low, thus requiring multiple rounds of amplification in PMCA as well as sample dilution to escape inhibitors [10,29,49,50]. Because RT-QuIC methodology offers the advantages of single cycle amplification, it offers potential for high through-put and reduced contamination risk-advantages for both detection and surveillance in the natural setting and in controlled longitudinal studies of prion pathogenesis and excretion. As estimated by the kinetics and magnitude of RT-QuIC responses, it appears that the concentration range of prions in saliva is approximately equivalent to 10^-6^ to 10^-8^ dilutions of CWD-positive brain. These results reinforce previous studies indicating that RT-QuIC can estimate the relative concentrations of infectious prions in a given sample [34].

Bioassay experiments also indicate that the levels of infectious prions in deer and sheep saliva are similar to near end-point dilutions of prion-infected brain [13,26]. Although complicated by the presence of inhibitors, blood-borne prions have been demonstrated through substantial dilution prior to assay or use antibody capture from plasma to both concentrate seeding activity and escape inhibitors [12,29,30,50]. Conversely, cerebrospinal fluid has been shown to have relatively high levels of prion seeding activity [11,51]. Overall, therefore, the results we report here for saliva using RT-QuIC are consistent with studies thus far employing *in vitro* amplification methods.

However, much as with PMCA, comparing the relative amplification of saliva samples to brain samples or any other sample from tissue or body fluid has a number of caveats. For example, each sample source potentially contains distinct amplification inhibitors, enhancers, as well as other factors that likely influence reaction rate, magnitude, or lag phase. Nevertheless, the comparisons between *in vitro* seeding assays and in vivo infectivity assays increasingly support careful extrapolations.

In summary, RT-QuIC offers sensitivity with relatively rapid single cycle amplification, versatility in application to tissues, biologic fluids, and environmental samples, and potential to define co-factors and inhibitors of prion conversion.

## Supporting Information

Figure S1 **Western blot analysis of CWD(+) saliva inoculated Tg[CerPrP] 5037 mice. Lanes 1 and 18 show PrP^C^ form 10% mouse brain homogenates without PK digestion. Big arrowhead marks location of undigested PrP^C^.** Lanes 2-17 are PK digested brain homogenates from IC inoculated mice. Lanes 2-5 are brain homogenates from mice IC inoculated with obex from deer #133. Lanes 6-9 are brain homogenates from mice IC inoculated with saliva from deer #133. Lanes 10-13 are brain homogenates from mice IC inoculated with saliva from deer #144. Lanes 14-17 are brain homogenates from mice IC inoculated with saliva from deer #123 a mock infected CWD(-) deer.
(TIF)

Figure S2 **Brain section western blot analysis of deer tested preclinically for prions in saliva.** A. PrP^Res^ western blot analysis of deer with 100% of PTA RT-QuIC positive replicates. B. Western blot PrP^Res^ analysis of deer with less than 100% of PTA RT-QuIC positive replicates. PK was added to all samples except one CWD(-) brain sample. Antibody BAR-224 was used for detection. Layout of western blots is the same as in figure 6.
(TIF)

Table S1 **Summary of RT-QuIC results. Comparison of RT-QuIC results from dilution or PTA experiments performed in laboratories at CSU and RML.**
(DOCX)
