# Supplementary figures and images for: Rapid Antemortem Detection of CWD Prions in Deer Saliva
Source: PLoS One. 2013 Sep 11;8(9):e74377. doi: 10.1371/journal.pone.0074377 (PMC3770611; doi:10.1371/journal.pone.0074377)

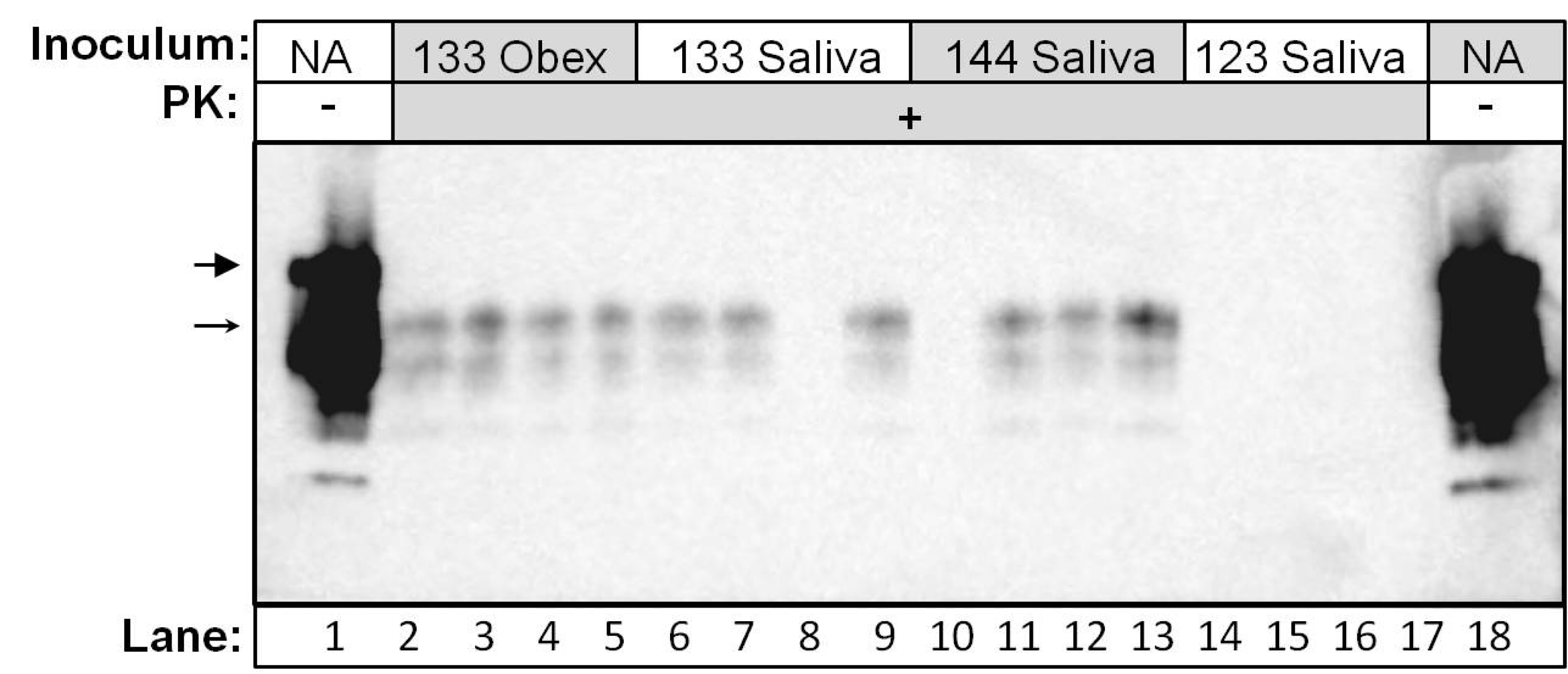

Supplement: Figure S1 — Western blot analysis of CWD(+) saliva inoculated Tg[CerPrP] 5037 mice. Lanes 1 and 18 show PrPC form 10% mouse brain homogenates without PK digestion. Big arrowhead marks location of undigested PrPC. Lanes 2-17 are PK digested brain homogenates from IC inoculated mice. Lanes 2-5 are brain homogenates from mice IC inoculated with obex from deer #133. Lanes 6-9 are brain homogenates from mice IC inoculated with saliva from deer #133. Lanes 10-13 are brain homogenates from mice IC inoculated with saliva from deer #144. Lanes 14-17 are brain homogenates from mice IC inoculated with saliva from deer #123 a mock infected CWD(-) deer. (TIF) [file pone.0074377.s001.tif]

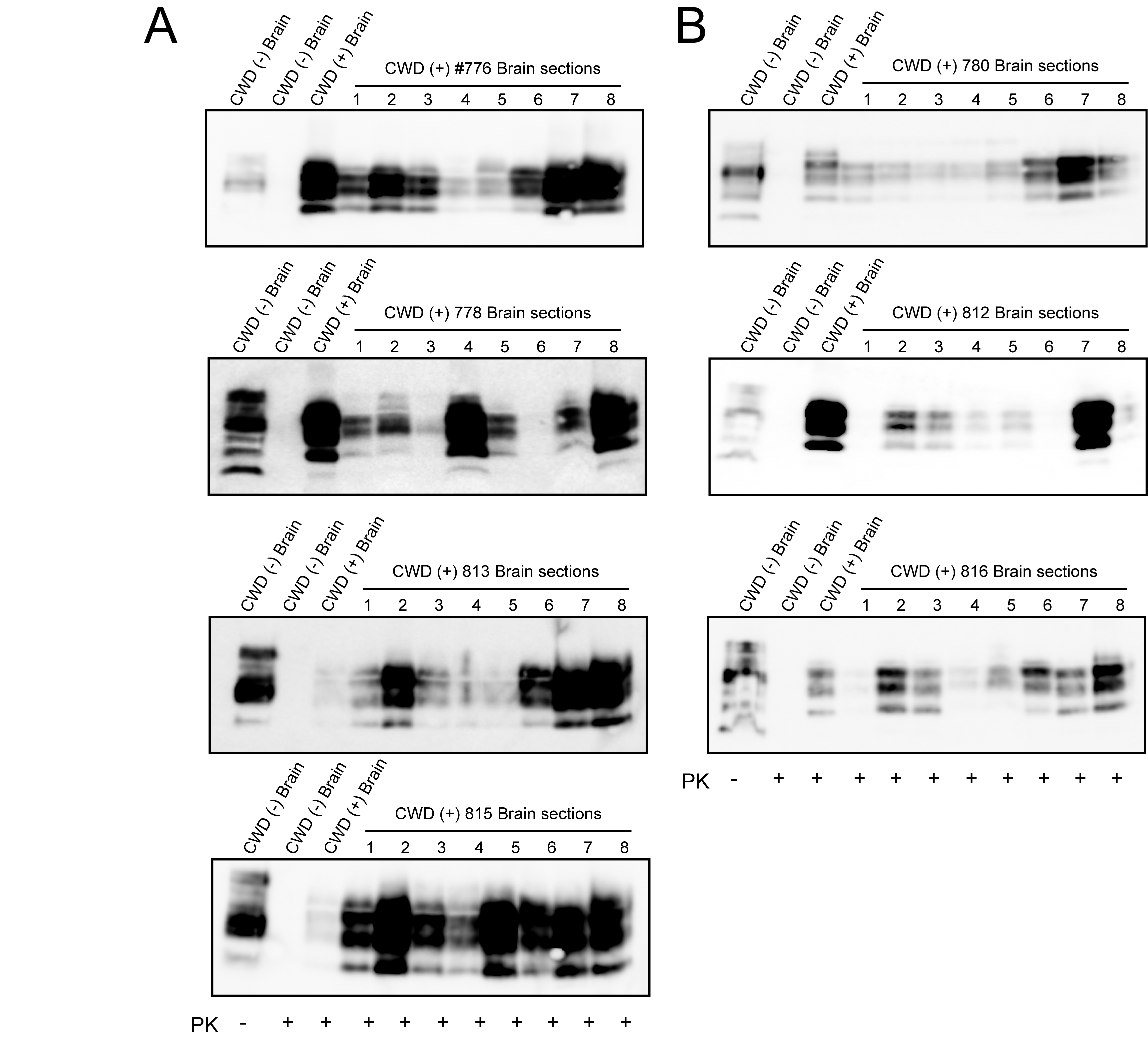

Supplement: Figure S2 — Brain section western blot analysis of deer tested preclinically for prions in saliva. A. PrPRes western blot analysis of deer with 100% of PTA RT-QuIC positive replicates. B. Western blot PrPRes analysis of deer with less than 100% of PTA RT-QuIC positive replicates. PK was added to all samples except one CWD(-) brain sample. Antibody BAR-224 was used for detection. Layout of western blots is the same as in figure 6. (TIF) [file pone.0074377.s002.tif]
