# Supplementary material for: Rapid Antemortem Detection of CWD Prions in Deer Saliva
Source: PLoS One. 2013 Sep 11;8(9):e74377. doi: 10.1371/journal.pone.0074377 (PMC3770611; doi:10.1371/journal.pone.0074377)
Supplement: Table S1 — Summary of RT-QuIC results. Comparison of RT-QuIC results from dilution or PTA experiments performed in laboratories at CSU and RML. (DOCX) [file pone.0074377.s003.docx]

**Supplemental Table 1:** RT-QuIC results from both laboratories are summarized. N.T. is not tested.

| **Summary of RT-QuIC results:** | | |  |  |
| --- | --- | --- | --- | --- |
|  | **CSU** | **CSU** | **RML** | **RML** |
| **Animal** | **Diluted** | **PTA** | **Diluted** | **PTA** |
| **Number** | **Saliva** | **Saliva** | **Saliva** | **Saliva** |
| 108 | 0/8 | 0/8 | 0/4 | 0/8 |
| 112 | 1/8 | 4/8 | 0/4 | 8/8 |
| 121 | 2/8 | 0/8 | N.T. | 8/8 |
| 132 | 0/8 | 1/8 | N.T. | 8/8 |
| 133 | 5/8 | 8/8 | N.T. | 8/8 |
| 136 | 0/8 | 2/8 | 1/4 | 6/8 |
| 137 | 0/8 | N.T. | N.T. | 4/8 |
| 138 | 3/8 | 5/8 | 4/4 | 10/12 |
| 143 | 0/8 | N.T. | 0/4 | 1/8 |
| 144 | 3/8 | 8/8 | N.T. | 8/8 |
| 773 | 0/8 | 0/8 | 0/4 | 3/8 |
| 775 | 1/8 | 1/8 | 0/4 | 0/8 |
| 776 | 0/8 | 7/8 | 0/4 | 8/8 |
| 777 | 1/8 | 0/8 | 0/4 | 2/12 |
| 778 | 1/8 | 5/8 | 0/4 | 12/12 |
| 780 | 0/8 | 3/8 | 0/4 | 1/8 |
| 781 | 0/8 | 0/8 | 0/4 | 0/8 |
| 785 | 1/8 | 3/8 | 0/4 | 12/12 |
| 812 | 1/8 | 1/8 | 0/4 | 0/8 |
| 813 | 0/8 | 1/8 | 0/4 | 16/16 |
| 815 | 1/8 | 3/8 | 0/4 | 8/8 |
| 816 | 3/8 | N.T. | N.T. | 0/8 |
| 817 | 1/8 | N.T. | N.T. | 6/8 |
| 818 | 1/8 | N.T. | N.T. | 2/8 |
| 810 | 0/8 | 0/8 | N.T. | 0/8 |
| 814 | 0/8 | 0/8 | N.T. | 0/8 |
| 819 | 0/8 | 0/8 | N.T. | 0/16 |
| 103 | 0/8 | N.T. | N.T. | 0/8 |
| 123 | 0/8 | N.T. | N.T. | 0/8 |
| 502 | 0/8 | 0/8 | 0/4 | 0/12 |
| 504 | 1/8 | 1/8 | 0/4 | 1/8 |
